# Microfibrillar‐associated protein 4 in serum is associated with asthma in Danish adolescents and young adults

**DOI:** 10.1002/iid3.254

**Published:** 2019-06-28

**Authors:** Benjamin Hoffmann‐Petersen, Raymond Suffolk, Jens Jakob Herrche Petersen, Thomas Houmann Petersen, Kirsten Arendt, Arne Høst, Susanne Halken, Grith Lykke Sorensen, Lone Agertoft

**Affiliations:** ^1^ Hans Christian Andersen Children's Hospital Odense University Hospital Odense Denmark; ^2^ Open Patient Data Explorative Network Odense University Hospital Odense Denmark; ^3^ Institute of Clinical Research, Faculty of Health Science University of Southern Denmark Odense Denmark; ^4^ Department of Pediatrics Hospital of Southern Jutland Aabenraa Denmark; ^5^ Department of Pediatrics Hospital of Southern Jutland Esbjerg Denmark; ^6^ Department of Pediatrics Hospital of Southern Jutland Kolding Denmark; ^7^ Institute of Molecular Medicine University of Southern Denmark Odense Denmark

**Keywords:** adolescent, asthma, biomarkers, child, microfibrillar‐associated protein 4

## Abstract

**Background:**

Microfibrillar‐associated protein 4 (MFAP4) is an extracellular matrix protein belonging to the fibrinogen‐related protein superfamily, which plays multifaceted roles in innate immunity and normal endothelial function. It has been proposed that MFAP4 promotes the development of asthma in vivo and proasthmatic pathways of bronchial smooth muscle cells in vitro. The aim of this study was to investigate the significance of serum MFAP4 in adolescents and young adolescents with persistent asthma.

**Methods:**

Prospective, observational study including adolescents and young adults (age 11‐27 years) previously diagnosed with asthma during childhood 2003 to 2005 (0‐15 years) at the four pediatric outpatient clinics in the Region of Southern Denmark (n = 449). Healthy controls were recruited at follow‐up (n = 314). Detection of serum MFAP4 was performed by AlphaLISA technique.

**Results:**

Current asthma was associated to a 14% higher mean level of serum MFAP4 compared with controls (exp*β* 1.14, 95% confidence intervals [CI], 1.05‐1.23) and a 6% higher mean level compared with subjects with no current asthma (exp*β* 1.06, 95% CI, 0.99‐1.13). No association was found at follow‐up between serum MFAP4 and self‐reported atopic symptoms (other than asthma), Asthma Control Test‐score, fractional exhaled nitric oxide (FeNO), nor to flow rate at 1 second, forced vital capacity, and forced expiratory flow 25% to 75%, response to short‐acting beta 2 agonist or mannitol.

**Conclusions:**

We found a significantly higher mean level of serum MFAP4 in adolescent and young adults with mild to moderate asthma compared with healthy controls but no association to FeNO and lung function nor to the response to short‐acting beta 2 agonist or mannitol. The result supports the hypothesis that MFAP4 plays a role in the pathogenesis of asthma although the marker did not demonstrate any obvious potential as an asthma biomarker in adolescents and young adults with asthma. To understand the possible proasthmatic functions of MFAP4, further investigation in specific asthma phenotypes and the underlying molecular mechanisms is warranted.

## 1 INTRODUCTION

Asthma is a common chronic airway disease characterized by recurrent episodes of respiratory symptoms such as wheeze, cough, dyspnea, and chest tightness caused by varying airway flow limitation.^1^ The pathogenesis of asthma is characterized by mucus‐cell hyperplasia, infiltration of inflammatory cells, and long‐term airway remodeling with increased deposition of connective tissue in the extracellular matrix, airway smooth muscle (ASM) hyperplasia and hypertrophy, and increased fibroblast and myofibroblast proliferation.^2^, ^3^, ^4^

The clinical assessment of asthma is based on airway symptoms, lung function, bronchial hyperreactivity, and indirect measures of airway inflammation, which especially in children give rise to some uncertainty about the nature of the underlying pathophysiological mechanisms in the airways.^1^ There is a need for objective markers that measure inflammation and remodeling in the asthmatic airway to ensure correct diagnostic and prognostic evaluation of children with airway symptoms suggestive of asthma.

Microfibrillar‐associated protein 4 (MFAP4) is an extracellular matrix protein belonging to the fibrinogen‐related protein superfamily, which plays multifaceted roles in innate immunity and normal endothelial function.^5^, ^6^, ^7^, ^8^ MFAP4 is expressed in elastin‐rich tissues such as inter‐alveolar walls and pulmonary arterioles where it is involved in elastic fiber formation by binding to tropoelastin.^9^, ^10^

MFAP4 has recently been shown to promote the development of asthma in vivo and display proasthmatic properties of bronchial smooth muscle cells in vitro and has been suggested to contribute to allergic asthma by promoting airway eosinophilia, airway hyperresponsiveness, and lung remodeling through regulation of ASM proliferation and eotaxin secretion in experimental studies.^11^

The objectives of this study were to investigate if the serum level of MFAP4 (sMFAP4) is associated with (a) asthma and (b) allergic sensitization in adolescents and young adults, and (c) whether sMFAP4 at the time of asthma diagnosis is associated with persistent asthma.

## 2 METHODS

### 2.1 Study design

In 2016 to 2017 we performed a prospective follow‐up on adolescents and young adults (age 11‐27 years) previously diagnosed with asthma during a 2‐year period from 2003 to 2005 (0‐15 years) at all four pediatric outpatient clinics in the Region of Southern Denmark covering 21% of the Danish population. Healthy controls with no medical history of asthma were recruited at follow‐up. Recruitment of controls was performed through a notice on the digital learning platforms on the same educational institutions as the origin of cases. The notice was addressed to parents if the subjects were below the age of 18 and addressed directly to subjects from 18 years of age.

### 2.2 Study population

During the baseline inclusion period from 1 October 2003 to 30 November 2005, a total of 1950 children aged 0 to 15 years suspected of asthma were referred to the pediatric departments by the primary care practitioners. Of those, 219 children were excluded due to language barriers or because they declined to participate. A total of 1015 children had the asthma diagnosis confirmed, 288 had the diagnosis rejected, and 428 were classified as inconclusive. Only children with a confirmed asthma diagnosis (n = 1015) were invited to participate in the follow‐up examination. A total of 44.2% (449/1015) subjects participated in the follow‐up examination. Of the 449 subjects included, 196 were classified as having current asthma and 253 as having no current asthma. A descriptive comparison of included subjects and dropouts is available on the online Supporting Information (Table SE1). At follow‐up 314 healthy controls with no medical history of asthma were included. Blood samples were obtained in 98.7% (753/763) and spirometry and reversibility test performed in 99.6% (760/763) of the participants. The mannitol test was only performed in subjects with a prior history of asthma in whom 94.4% (424/449) had a conclusive test performed. The analytical sample comprised those in whom the conclusive asthma classification and serum measurements were available (n = 752).

### 2.3 Baseline data collection at the time of diagnosis

Baseline data were extracted from a structured database and biobank established during the baseline examination. The data were collected by the treating staff and included a structured questionnaire‐based interview (regarding atopic predisposition, medical history, use of medication, and environmental factors), clinical examination by a pediatrician, blood sampling, and spirometry. Reversibility and exercise tests were performed when appropriate.

### 2.4 Follow‐up data collection

The follow‐up examination was performed by the research team, including experienced medical doctors and pediatric nurses with a specialty within the field of pediatric asthma and allergology. Each participant completed a structured questionnaire‐based interview (regarding atopic predisposition, medical history, use of medication, and environmental factors), clinical examination, blood and urine sampling, measurement of fractional exhaled nitric oxide (FeNO), spirometry with reversibility test, mannitol provocation test, and dual‐energy *x*‐ray absorptiometry. The subjects completed questionnaires regarding asthma control, Asthma Control Test (ACT), and quality of life (Asthma Quality of Life Questionnaire).^12^, ^13^ Controls completed the same examination program as the cases except for the mannitol provocation test and asthma questionnaires.

### 2.5 Definition of asthma

Current asthma was defined by recurrence or persistence of at least two of three symptoms: cough, wheeze, and shortness of breath (not triggered only by infection) within the last 12 months and at least one of the following: positive bronchodilator reversibility test (salbutamol) and/or positive mannitol test. Furthermore, subjects were defined as having asthma if they had a history of symptoms and daily use of, inhaled corticosteroids (ICS), fixed combination of ICS, and long‐acting beta 2 agonist (LABA) or classical exercise‐induced asthma symptoms and use of inhaled beta2‐agonists when needed.

### 2.6 Spirometry

Lung function was measured by spirometry (SpiroUSB; CareFusion Ltd, United Kingdom). Reversibility test was performed with salbutamol (Buventol Easyhaler, Orion Pharma A/S, Denmark) and mannitol provocation testing (Osmohale; Pharmaxis Ltd, Australia). All measurements were performed according to generally accepted methods and criteria.^14^, ^15^ Parameters obtained for statistical analysis at follow‐up were forced expiratory flow rate at 1 second (FEV1), forced vital capacity (FVC), and forced expiratory flow (FEF 25%‐75%). At baseline FEV1, FVC, expiratory flow at mid‐range expiration (25%‐75%), and peak flow rate were obtained. Predicted values were calculated using the multiethnic reference values for spirometry developed by the European Respiratory Society Task Force.^16^ FeNO was measured before spirometry using NIOX VERO, Circassia Pharmaceuticals plc, United Kingdom.

### 2.7 Serum sampling

Venous blood samples were drawn before lung function measurements, collected in dry tubes and allowed to clot for a minimum of 30 minutes. The samples were centrifuged for 10 minutes at 2000*g* at room temperature. The serum layer was pipetted and stored at −80°C until laboratory analysis.

### 2.8 Serum MFAP4

Detection of sMFAP4 was performed by the AlphaLISA technique using 384‐well microtiter plates. The analysis was performed in duplicates and sample covariance was accepted if ≤10%. A detailed description of the method used to determine sMFAP4 concentrations has been described elsewhere.^9^

### 2.9 Allergic sensitization

Specific immunoglobulin e (s‐IgE) was measured quantitatively using single ImmunoCAP after an initial qualitative screening with ImmunoCap Phadiatop (Thermo Fisher Diagnostics Aps, Denmark). The samples were tested to specific IgE with five food allergens (milk, egg, peanut, wheat, and soy) and 10 inhalant allergens (birch, timothy grass, mugwort, horse, dog, cat, dermatophagoides pteronyssinus, dermatophagoides farinae, alternaria, and cladosporium). Sensitization was defined as s‐IgE ≥0.35 kU/L.

### 2.10 Statistical analysis

The distribution of data was assessed visually using histograms and QQ‐plots. Due to the non‐normal distribution of observations of sMFAP4, the approximation to normal distribution was achieved by logarithmic transformation (the natural logarithm, *ln*) before further statistical analysis.

sMFAP4 levels, stratified by current asthma, no current asthma, and controls, are presented as geometric mean and 95% confidence intervals (CI). Ordinary least square regression was used to test differences between groups and to estimate associations between *ln* sMFAP4 as the outcome and relevant exposure variables. An initial *F*‐test was used to test the overall difference between coefficients followed by pairwise comparison of estimates. The univariate and adjusted associations between sMFAP4 and relevant exposure variables are presented by back‐transformed coefficients (e^ln(β)^) and CI (95%) with associated *P* values. Logistic regression was used to evaluate the association between baseline sMFAP4 and current asthma. All calculations were performed using complete case analysis. The level of significance was set to 0.05. Stata/IC 15.1 (StataCorp) was used for all analysis.

### 2.11 Covariates

The data were analyzed using three models to accommodate potential confounders. Model 1 included age and sex as covariates. Model 2 included age, sex, BMI, and current smoking. Model 3 included age, sex, BMI, current smoking, and sensitization to any inhalant allergen. When the association between sMFAP4 and allergic sensitization was analyzed model 3 included age, sex, BMI, current smoking, and asthma classification at follow‐up.

### 2.12 Ethics

The protocol was approved by the Regional Scientific Ethical Committee of Southern Denmark (S‐20120093) and the Danish Data Protection Agency (95 to 50819) and was conducted in accordance with the Helsinki Declaration. Informed consent was obtained from each participant and from the parents of participants below 18 years of age.

## 3 RESULTS

### 3.1 Population characteristics

Basic characteristics among the groups (current asthma, no current asthma, and controls) are present in Table 1. The groups had similar distributions of current smokers, number of siblings, and ethnic origin but significant differences according to age, sex, BMI, atopic disposition and comorbidity, ACT‐score, and antiasthmatic treatment.

**Table 1:** Characteristics and potential confounders measured at follow‐up according to asthma classification at follow‐up: controls, no current asthma, and current asthma

	Controls	No current asthma	Current asthma	
	(n = 314)	(n = 253)	(n = 196)	*χ* ^2^/ANOVA
	% (cases/total)	% (cases/total)	% (cases/total)	*P* value
Basic characteristics				
Sex‐ female	61.8 (194/314)	39.9 (101/253)	45.9 (90/196)	<0.001
Age‐ mean (sd)	18.4 (4.5)	17.6 (4.4)	18.7 (4.1)	0.030
BMI‐ mean (sd)	22.2 (3.8)	22.9 (4.7)	23.3 (5.7)	0.018
Ethnicity‐ caucasian	98.1 (308/314)	96.4 (244/253)	95.9 (188/196)	0.312
Parental asthma^a^	10.5 (33/314)	29.6 (75/253)	33.2 (65/196)	<0.001
No of siblings	1.4 (0.9)	1.3 (0.8)	1.4 (0.9)	0.244
Active smoking	5.4 (17/314)	8.7 (22/253)	10.7 (21/196)	0.080
Symptom score (Asthma Control Test)^b^				
Well‐controlled (score >20)	–	94.0 (236/251)	77.4 (151/195)	
Uncontrolled (score ≤20)	–	6.0 (15/251)	22.6 (44/195)	
Current atopic symptoms^c^				
Hayfever	2.9 (9/314)	44.7 (113/253)	74.5 (146/196)	<0.001
Eczema	2.2 (7/314)	11.1 (28/253)	27.7 (54/195)	<0.001
Food allergy	1.0 (3/314)	5.1 (13/253)	10.2 (20/196)	<0.001
Urticaria	0.6 (2/314)	4.3 (11/253)	13.8 (27/196)	<0.001
Current medication				
No treatment	–	86.8 (217/250)	11.3 (22/194)	
SABA only	–	13.2 (33/250)	20.1 (39/194)	
ICS low dose	–	–	29.4 (57/194)	
ICS low dose + LABA	–	–	29.9 (58/194)	
ICS high dose + LABA	–	–	9.3 (18/194)	
Add on (thiotropium/biologicals)	–	–	–	
Allergic sensitization				
Inhalant allergens	18.8 (58/309)	54.5 (134/246)	78.1 (153/196)	<0.001
Food allergens	2.6 (8/309)	10.6 (26/246)	16.3 (32/196)	<0.001

Abbreviations: ACT, Asthma Control Test; ANOVA**,** analysis of variance; ICS, inhalant corticosteroids; LTRA, leukotrien receptor antagonists; SABA, short‐acting beta2‐agonists; sd, standard deviation.

^a^ Medical history of asthma in ≥1 parent.

^b^ Not filled in by controls.

^c^ Self‐reported symptoms.

### 3.2 Associations between sMFAP4 and potential confounders

Serum MFAP4 showed a significant association with both age (exp*β* 1.02, 95% CI, 1.01‐1.02) and female sex (exp*β* 1.10, 95% CI, 1.04‐1.16) (Table 2). Current smoking was associated with a slightly lower level of sMFAP4, though not significant in the univariate analysis, whereas adjustment for age made the association stronger (exp*β* 0.86, 95% CI, 0.78‐0.95). No association was observed between sMFAP4 and body mass index and ethnic origin (Table 2).

**Table 2:** Association between serum MFAP4 and potential confounders

	Univariate analysis		Multivariate analysis	
	(n = 752)		(n = 752)	
	exp*β* (95% CI)	*P* value	exp*β* (95% CI)	*P* value
Age	1.02 (1.01; 1.02)	<0.001	1.02 (1.01; 1.02)	<0.001
Sex‐female	1.10 (1.04; 1.16)	0.001	1.09 (1.03; 1.15)	0.002
BMI	1.00 (1.00; 1.01)	0.313	0.99 (0.99; 1.00)	0.043
Etnicity‐ Caucasian	1.00 (0.85; 1.17)	0.991	0.98 (0.84; 1.14)	0.773
Current smoking	0.92 (0.84; 1.02)	0.124	0.86 (0.78; 0.95)	0.003

*Note*: The results are shown as the back‐transformed coefficients e^ln(β)^, 95% confidence intervals and *P* values. The multivariate model is adjusted for age, sex, and asthma classification at follow‐up.

Abbreviations: CI, confidence interval; MFAP4, microfibrillar‐associated protein 4

### 3.3 Serum MFAP4 is associated with current asthma

**Figure 1:**
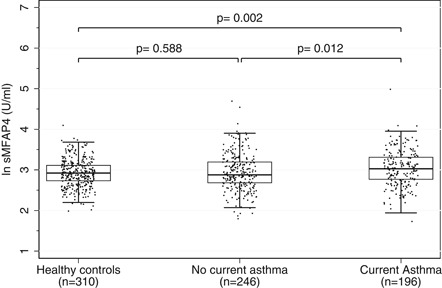
Boxplot presenting log‐transformed serum levels of MFAP4 (unadjusted) measured at follow‐up stratified by asthma classification at follow‐up: controls, no current asthma, and current asthma. MFAP4, microfibrillar‐associated protein 4

All observations stratified by classification at follow‐up are present in Figure 1. The unadjusted analysis showed a 12% higher mean level of sMFAP4 in subjects with current asthma compared with controls (exp*β* 1.12, 95% CI, 1.04‐1.19) and a tendency towards a higher mean level compared with subjects with no current asthma (exp*β* 1.02, 95% CI, 0.96‐1.08) (Table 3). Based on the findings regarding potential confounders, we adjusted the analysis for age and sex, BMI, current smoking, and allergic sensitization to any inhalant allergen. The multivariate analysis showed a strengthened association with a 14% higher mean level of sMFAP4 in subjects with current asthma compared with controls (exp*β* 1.14, 95% CI, 1.05‐1.23). No significant difference between subjects with no current asthma and controls was found. The association with current asthma was only present in the upper quartiles of sMFAP4 (Table SE2). We found no association between sMFAP4 at follow‐up and self‐reported atopic symptoms other than asthma, ACT‐score, FeNO nor to FEV1, FVC, FEF 25% to 75%, response to short‐acting beta 2 agonist or mannitol (Table 4). Correspondingly, we found no association between baseline sMFAP4 and atopic symptoms and spirometry performed at baseline (Table 5).

**Table 3:** Association between sMFAP4 and asthma at follow‐up

	Univariate analysis		Model 1		Model 2		Model 3	
	(n = 752)		(n = 752)		(n = 752)		(n = 751)	
	exp*β* (95% CI)	*P* value	exp*β* (95% CI)	*P* value	exp*β* (95% CI)	*P* value	exp*β* (95% CI)	*P* value
Controls	1.00 (Ref.)	–	1.00 (Ref.)	–	1.00 (Ref.)	–	1.00 (Ref.)	–
No current asthma	1.02 (0.96‐1.08)	0.588	1.05 (0.98‐1.12)	0.138	1.06 (1.00‐1.13)	0.066	1.06 (0.99‐1.13)	0.094
Current asthma	1.12 (1.04‐1.19)	0.002	1.13 (1.05‐1.20)	<0.001	1.14 (1.07‐1.22)	<0.001	1.14 (1.05‐1.23)	0.001

*Note*: Subjects with current asthma and no current asthma are compared to controls. The results are shown as the back‐transformed coefficients e^ln(β)^, 95% confidence intervals and *P* values. Model 1 is adjusted for age and sex. Model 2 is adjusted for age, sex, BMI, and current smoking. Model 3 is adjusted for age, sex, BMI, current smoking, and sensitization to any inhalant allergen.

Abbreviations: CI, confidence interval; MFAP4, microfibrillar‐associated protein 4; sMFAP4, serum level of MFAP4

**Table 4:** Association between sMFAP4 at follow‐up and spirometry, airway response to short‐acting β2‐agonist and mannitol, fractional exhaled nitric oxide (FeNO) and allergic sensitization measured at follow‐up

	Univariate analysis		Model 1		Model 2		Model 3	
	exp*β* (95% CI)	*P* value	exp*β* (95% CI)	*P* value	expβ (95% CI)	*P* value	exp*β* (95% CI)	*P* value
Spirometry (% of predicted)								
FEV1	0.91 (0.72; 1.16)	0.443	0.83 (0.65; 1.04)	0.111	0.81 (0.64; 1.03)	0.081	0.90 (0.71; 1.15)	0.405
FVC	1.08 (0.84; 1.39)	0.557	0.98 (0.76; 1.25)	0.853	0.98 (0.76; 1.25)	0.867	0.97 (0.76; 1.25)	0.833
FEF 25%‐75%	0.93 (0.82; 1.06)	0.294	0.89 (0.78; 1.01)	0.066	0.88 (0.77; 1.00)	0.043	0.95 (0.83; 1.08)	0.438
Airway responsiveness								
Reversibility test	1.56 (0.90; 2.71)	0.112	1.84 (1.07; 3.15)	0.028	1.90 (1.11; 3.26)	0.019	1.51 (0.86; 2.65)	0.155
Mannitol test (positive)^a^	1.06 (0.96; 1.17)	0.262	1.04 (0.94; 1.14)	0.435	1.04 (0.94; 1.14)	0.433	1.00 (0.90; 1.11)	0.974
Eosinophilic inflammation								
lnFeNO	1.04 (1.00; 1.08)	0.082	1.03 (0.99; 1.07)	0.141	1.03 (0.98; 1.07)	0.221	1.00 (0.96; 1.04)	0.993
Allergic sensitization								
Inhalant allergens	1.06 (1.00; 1.11)	0.053	1.04 (0.99; 1.10)	0.118	1.05 (1.00; 1.11)	0.071	1.00 (0.94; 1.07)	0.925
Food allergens	1.00 (0.91; 1.11)	0.940	1.03 (0.94; 1.14)	0.494	1.03 (0.94; 1.14)	0.481	1.00 (0.91; 1.10)	0.991

*Note*: The results are shown as the back‐transformed coefficients e^ln(β)^, 95% confidence intervals and *P* values. Model 1 is adjusted for age and sex. Model 2 is adjusted for age, sex, BMI, and current smoking. Model 3 is adjusted for age, sex, BMI, current smoking, and asthma classification at follow‐up.

Abbreviations: FeNO, fractional exhaled nitric oxide; FEF, forced expiratory flow; FEV1, forced expiratory volume in 1 second; FVC, forced vital capacity; SABA, short‐acting beta2‐agonists.

^a^ Mannitol test only performed in subjects with previous or current asthma.

**Table 5:** Association between sMFAP4 at baseline and spirometry, blood eosinophils, total IgE, and allergic sensitization measured at baseline

	Univariate analysis		Model 1		Model 2		Model 3	
	exp*β* (95% CI)	*P* value	exp*β* (95% CI)	*P* value	exp*β* (95% CI)	*P* value	exp*β* (95% CI)	*P* value
Atopic symptoms								
Hayfever	0.96 (0.85; 1.09)	0.518	1.02 (0.89; 1.16)	0.816	1.00 (0.87; 1.14)	0.971	1.00 (0.88; 1.14)	0.992
Eczema	0.95 (0.84; 1.08)	0.439	0.96 (0.85; 1.09)	0.546	0.98 (0.87; 1.10)	0.696	0.95 (0.84; 1.07)	0.426
Urticaria	0.93 (0.75; 1.17)	0.549	0.96 (0.77; 1.21)	0.752	0.95 (0.76; 1.19)	0.680	0.95 (0.76; 1.19)	0.634
Food allergy	0.92 (0.73; 1.14)	0.439	0.93 (0.74; 1.16)	0.498	0.92 (0.74; 1.14)	0.434	0.92 (0.74; 1.15)	0.452
Spirometry (% of predicted)								
FEV1	1.00 (0.99; 1.00)	0.099	0.99 (0.99; 1.00)	0.037	0.99 (0.99; 1.00)	0.068	1.00 (0.99; 1.00)	0.097
FVC	1.00 (0.99; 1.00)	0.892	1.00 (0.99; 1.00)	0.823	1.00 (0.99; 1.01)	0.943	1.00 (0.99; 1.01)	0.978
FMEF	1.00 (0.99; 1.00)	0.147	1.00 (0.99; 1.00)	0.082	1.00 (0.99; 1.00)	0.118	1.00 (0.99; 1.00)	0.179
Peak flow	1.00 (0.99; 1.00)	0.664	1.00 (0.99; 1.00)	0.605	1.00 (0.99; 1.01)	0.935	1.00 (0.99; 1.01)	0.920
Eosinophilic inflammation								
Eosinophils	1.00 (0.99; 1.00)	0.281	1.00 (0.99; 1.00)	0.250	1.00 (0.99; 1.00)	0.270	1.00 (0.99; 1.00)	0.198
Allergic sensitization								
Total IgE	1.00 (1.00; 1.00)	0.465	1.00 (1.00; 1.00)	0.940	1.00 (1.00; 1.00)	0.937	1.00 (1.00; 1.00)	0.622
Inhalant allergens	0.92 (0.82; 1.04)	0.178	0.97 (0.85; 1.10)	0.630	0.98 (0.86; 1.12)	0.814	0.93 (0.81; 1.06)	0.262
Food	1.14 (1.01; 1.29)	0.032	1.16 (1.03; 1.31)	0.018	1.17 (1.04; 1.32)	0.012	1.14 (1.01; 1.29)	0.035

The results are shown as the back‐transformed coefficients e^ln(β)^, 95% confidence intervals and *P* values. Model 1 is adjusted for age and sex. Model 2 is adjusted for age, sex, and current exposure to tobacco smoke. Model 3 is adjusted for age, sex, current exposure to tobacco smoke, and asthma classification at follow‐up.

Abbreviations: FeNO, fractional exhaled nitric oxide; FEV1, forced expiratory volume in 1  second; FMEF, expiratory flow at mid‐range expiration; FVC, forced vital capacity; MFAP4, microfibrillar‐associated protein 4; PEFR, peak flow rate; sMFAP4, serum level of MFAP4; SABA, short‐acting beta2‐agonists; IgE: immunoglobulin e.

### 3.4 The association between sMFAP4 and allergic sensitization

We found an association between sMFAP4 at follow‐up and sensitization to inhalant allergens. The association was robust to adjustment for sex, age, and current smoking but the association diminished when we included classification at follow‐up in the model (Table 4). By stratification according to asthma classification, we found a uniform tendency towards a higher sMFAP4 level in sensitized subjects in all three groups although the association was nonsignificant (Table E5). Baseline sMFAP4 was associated with sensitization to food allergens but not to inhalant allergens, although the effect was markedly reduced when asthma classification at follow‐up was included as a covariate (Table 5). Lastly, we found no correlation between sMFAP4 and the level of s‐IgE in subjects with s‐IgE ≥0.35 U/mL (Figures SE4 and SE5).

### 3.5 Serum MFAP4 measured at baseline is not associated with current asthma at follow‐up

Serum MFAP4 from baseline samples was analyzed with asthma (yes/no) as outcome using logistic regression and showed no association to current asthma. Subsequently, multivariate linear regression was applied to analyze the association between baseline sMFAP4 in relation to the same clinical parameters described above which also showed no association to symptoms, lung function, or sensitization at follow‐up.

## 4 DISCUSSION

### 4.1 Main findings

In the present study, we investigated the significance of serum MFAP4 in relation to asthma in adolescents and young adults and found a significantly higher mean level of sMFAP4 in adolescents and young adults with current asthma compared with healthy controls. We found an ambiguous association between sMFAP4 and allergic sensitization that diminished in the multivariate model including asthma classification at follow‐up. We observed no association to FeNO and lung function nor to the response to short‐acting beta 2 agonist or mannitol. Also, we found no longitudinal association between sMFAP4 measured at the time of asthma diagnosis and persistence of asthma at follow‐up.

### 4.2 Interpretation of the findings

To our knowledge, this is the first clinical study investigating the association between MFAP4 and asthma in adolescents and young adults. Our main result supports previous in vitro and in vivo studies investigating the possible role of MFAP4 in allergic asthma. Pilecki et al^11^ demonstrated elevated levels of sMFAP4 in ovalbumin and HDM treated mice in an allergic asthma model, and that MFAP4 is upregulated in asthmatic bronchial smooth muscle cells promoting smooth muscle cell proliferation and migration by interactions with the *α*_1_ *β*_3_‐integrin receptor.^17^ In a population including patients with chronic obstructive lung disease, Johansson et al^18^ found that plasma levels of MFAP4 increased with disease severity and in the months following an acute exacerbation.^19^

It can be speculated that our observed association between sMFAP4 and current asthma is a result of structural changes in the extracellular matrix including alterations in MFAP4 expression and that the asthmatic airway inflammation subsequently induces enhanced leakage of MFAP4 from the distal airways into the circulation. Opposed to this hypothesis is the lack of association to lung function and bronchodilator response and airway hyperresponsiveness that previously has been shown to be associated with airway remodeling, including ASM hypertrophy and hyperplasia, in children and adults with asthma.^20^, ^21^ Previously, it has been established that airway remodeling, which includes increased reticular basement membrane thickness and increased ASM, is already present during early life in children with moderate to severe asthma.^22^, ^23^, ^24^, ^25^ An explanation of the lack of association to the clinical measures of asthma might be the characteristics of our study population, which mainly included subjects with mild to moderate asthma, in whom chronic ASM changes would not be expected, and that the increased level of MFAP4 is due to subclinical extracellular matrix (ECM) remodeling. Less than five percent of our subjects with current asthma had severe asthma defined as poor control despite maximal maintenance therapy (GINA step 4) and acceptable adherence to treatment. Also, we have to consider the risk of residual confounding despite our effort to include relevant covariates in the models. The nature of the present study does not allow us to conclude on the underlying cellular mechanisms nor to establish any causal relationship between MFAP4 and asthma.

### 4.3 Strengths and limitations

A major strength of this study is the large study population including comprehensively characterized subjects in a real‐life clinical setting. We have identified and included relevant confounders although the literature on potential confounding factors is sparse. An important limitation of our study is the lack of bronchoalveolar lavage fluid and bronchial biopsy samples, which limits our ability to scrutinize the underlying molecular mechanisms of our results. Future clinical studies on MFAP4 should focus on more specific asthma phenotypes such as severe persistent asthma where we would expect a higher degree of structural changes in the extracellular matrix. Furthermore, it would be relevant to explore to what extent the variation in sMFAP4 is explained by common variations in the MFAP4 gene and to investigate associations to MFAP4 messenger RNA expression and Th2 specific cytokine responses in the target tissue.

## 5 CONCLUSION

We found a significantly higher mean level of serum MFAP4 in adolescent and young adults with mild to moderate asthma compared to healthy controls but no association to FeNO and lung function nor to the response to short‐acting beta 2 agonist or mannitol. The result supports the hypothesis that MFAP4 plays a role in the pathogenesis of asthma although the marker did not demonstrate any obvious potential as an asthma biomarker in adolescents and young adults with asthma. To understand the possible proasthmatic functions of MFAP4, further investigation in specific asthma phenotypes and the underlying molecular mechanisms is warranted.

## CONFLICT OF INTERESTS

Grith L Sorensen owns two patents P1389DK00 and P1183DK00 regarding MFAP4 binding antibodies blocking the interaction between MFAP4 and integrin receptors. The remaining authors declare that they have no conflict of interests.

## AUTHOR CONTRIBUTIONS

SH and AH were responsible for the baseline study as a whole from design to the conduction of the study and data collection. LA, BH, SH, and AH designed and initiated the follow‐up study. BH, RA, JJP, KA, and THP performed clinical examinations, data collection, and serum sampling. BH was responsible for the uniformity and consistency of the study across the hospitals and supervised all examinations. GLS was responsible for laboratory analysis of MFAP4. BH was responsible for data collection, data analysis, and drafting the manuscript. All coauthors have contributed substantially to the interpretation of the data, providing crucial intellectual input, and approval of the final draft of the manuscript.

## Supporting information

Supporting information

## Data Availability

The standard terms for research projects and the Danish Act on Processing of Personal Data define the rules of data sharing and will be followed. Data used for the manuscript may be obtained in anonymous form after application for permission to the regional Danish Data Protection Agency (https://www.datatilsynet.dk/english).
