# Supplementary material for: Microfibrillar‐associated protein 4 in serum is associated with asthma in Danish adolescents and young adults
Source: Immun Inflamm Dis. 2019 Jun 28;7(3):150–9. doi: 10.1002/iid3.254 (PMC6688087; doi:10.1002/iid3.254)
Supplement: Supplementary file 1 — Supporting information [file IID3-7-150-s001.docx]

**Supporting information**

**Table E1.** Descriptive dropout analysis. Distribution of basic characteristics, atopic symptoms, use of anti-asthmatic medication and allergic sensitization among subjects included at follow-up and subjects lost to follow-up.

**Table E2.** Association between quartiles of sMFAP4 and current asthma at follow-up. The results are shown as the backtransformed coefficients e^ln(β)^, 95% confidence intervals and p-values.

**Figure E3** Scatterplots presenting sMFAP4 at follow-up by specific IgE in serum to single inhalant allergens at follow-up. Only data from subjects with a positive Phadiatop screening is presented.

**Figure E4** Scatterplots presenting sMFAP4 at baseline by specific IgE in serum to single food allergens at baseline.

**Table E5.** Association between sMFAP4 and sensitization to groups of allergens at follow-up stratified by classification at follow-up: *controls*, *no current asthma* and *current asthma*. The results are shown as the backtransformed coefficients e^ln(β)^, 95% confidence intervals and p-values.
